# Incidence of typhoid and paratyphoid fever in Bangladesh, Nepal, and Pakistan: results of the Surveillance for Enteric Fever in Asia Project

**DOI:** 10.1016/S2214-109X(22)00119-X

**Published:** 2022-06-14

**Authors:** Denise O Garrett, Ashley T Longley, Kristen Aiemjoy, Mohammad T Yousafzai, Caitlin Hemlock, Alexander T Yu, Krista Vaidya, Dipesh Tamrakar, Shampa Saha, Isaac I Bogoch, Kashmira Date, Senjuti Saha, Mohammad Shahidul Islam, K M Ishtiaque Sayeed, Caryn Bern, Sadia Shakoor, Irum F Dehraj, Junaid Mehmood, Mohammad S I Sajib, Maksuda Islam, Rozina S Thobani, Aneeta Hotwani, Najeeb Rahman, Seema Irfan, Shiva R Naga, Ashraf M Memon, Sailesh Pradhan, Khalid Iqbal, Rajeev Shrestha, Hafizur Rahman, Md Mahmudul Hasan, Saqib H Qazi, Abdul M Kazi, Nasir S Saddal, Raza Jamal, Mohammed J Hunzai, Tanvir Hossain, Florian Marks, Alice S Carter, Jessica C Seidman, Farah N Qamar, Samir K Saha, Jason R Andrews, Stephen P Luby

**Affiliations:** a Applied Epidemiology, Sabin Vaccine Institute, Washington, DC, USA; b National Foundation for the Centers for Disease Control and Prevention, Atlanta, GA, USA; c Global Immunization Division, Centers for Disease Control and Prevention, Atlanta, GA, USA; d Department of Public Health Sciences, University of California Davis, Davis, CA, USA; e Division of Infectious Diseases and Geographic Medicine, School of Medicine, Stanford University, Stanford, CA, USA; f Department of Pediatrics and Child Health, Aga Khan University, Karachi, Pakistan; g Department of Pathology and Laboratory Medicine, Aga Khan University, Karachi, Pakistan; h Department of Surgery, Aga Khan University, Karachi, Pakistan; i Dhulikhel Hospital, Kathmandu University Hospital, Kavrepalanchok, Nepal; j Child Health Research Foundation, Dhaka, Bangladesh; k Department of Medicine, University of Toronto, Toronto, ON, Canada; l Department of Epidemiology and Biostatistics, University of California San Francisco, San Francisco, CA, USA; m Kharadar General Hospital, Karachi, Pakistan; n Kathmandu Medical College Teaching Hospital, Kathmandu, Nepal; o National Institute of Child Health, Karachi, Pakistan; p Maternal and Child Health Division, icddr,b, Dhaka, Bangladesh; q International Vaccine Institute, Seoul, South Korea; r Cambridge Institute of Therapeutic Immunology and Infectious Disease, University of Cambridge School of Clinical Medicine, Cambridge, UK; s University of Antananarivo, Antananarivo, Madagascar; t Heidelberg Institute of Global Health, University of Heidelberg, Heidelberg, Germany; u Bangladesh Institute of Child Health, Dhaka Shishu Hospital, Dhaka, Bangladesh

## Abstract

**Background:**

Precise enteric fever disease burden data are needed to inform prevention and control measures, including the use of newly available typhoid vaccines. We established the Surveillance for Enteric Fever in Asia Project (SEAP) to inform these strategies.

**Methods:**

From September, 2016, to September, 2019, we conducted prospective clinical surveillance for *Salmonella enterica* serotype Typhi (*S* Typhi) and Paratyphi (*S* Paratyphi) A, B, and C at health facilities in predetermined catchment areas in Dhaka, Bangladesh; Kathmandu and Kavrepalanchok, Nepal; and Karachi, Pakistan. Patients eligible for inclusion were outpatients with 3 or more consecutive days of fever in the last 7 days; inpatients with suspected or confirmed enteric fever; patients with blood culture-confirmed enteric fever from the hospital laboratories not captured by inpatient or outpatient enrolment and cases from the laboratory network; and patients with non-traumatic ileal perforation under surgical care. We used a hybrid surveillance model, pairing facility-based blood culture surveillance with community surveys of health-care use. Blood cultures were performed for enrolled patients. We calculated overall and age-specific typhoid and paratyphoid incidence estimates for each study site. Adjusted estimates accounted for the sensitivity of blood culture, the proportion of eligible individuals who consented and provided blood, the probability of care-seeking at a study facility, and the influence of wealth and education on care-seeking. We additionally calculated incidence of hospitalisation due to typhoid and paratyphoid.

**Findings:**

A total of 34 747 patients were enrolled across 23 facilitates (six tertiary hospitals, surgical wards of two additional hospitals, and 15 laboratory network sites) during the study period. Of the 34 303 blood cultures performed on enrolled patients, 8705 (26%) were positive for typhoidal *Salmonella*. Adjusted incidence rates of enteric fever considered patients in the six tertiary hospitals. Adjusted incidence of *S* Typhi, expressed per 100 000 person-years, was 913 (95% CI 765–1095) in Dhaka. In Nepal, the adjusted typhoid incidence rates were 330 (230–480) in Kathmandu and 268 (202–362) in Kavrepalanchok. In Pakistan, the adjusted incidence rates per hospital site were 176 (144–216) and 103 (85–126). The adjusted incidence rates of paratyphoid (of which all included cases were due to *S* Paratyphi A) were 128 (107–154) in Bangladesh, 46 (34–62) and 81 (56–118) in the Nepal sites, and 23 (19–29) and 1 (1–1) in the Pakistan sites. Adjusted incidence of hospitalisation was high across sites, and overall, 2804 (32%) of 8705 patients with blood culture-confirmed enteric fever were hospitalised.

**Interpretation:**

Across diverse communities in three south Asian countries, adjusted incidence exceeded the threshold for “high burden” of enteric fever (100 per 100 000 person-years). Incidence was highest among children, although age patterns differed across sites. The substantial disease burden identified highlights the need for control measures, including improvements to water and sanitation infrastructure and the implementation of typhoid vaccines.

**Funding:**

Bill & Melinda Gates Foundation.

## Introduction

Enteric fever, due to *Salmonella enterica* serotype Typhi (*S* Typhi) and Paratyphi (*S* Paratyphi) A, B, and C, is a preventable disease causing an estimated 14 million illnesses and 136 000 deaths worldwide in 2017.^1^ Illness is caused by ingesting faecally contaminated food or water. Although enteric fever is largely controlled in high-income countries through sanitation infrastructure improvements, in low-income and middle-income countries the disease is largely managed with antibiotics.^2^, ^3^ The spread of antimicrobial resistance is undermining this management approach, however, as increasing drug resistance makes enteric fever more difficult and expensive to treat.^4^, ^5^

**Research in context**

**Evidence before this study**

We searched PubMed, medRxiv, and SSRN for articles available as of Feb 23, 2022, with no date or language restrictions, using the search terms “(typhoid OR Salmonella Typhi OR Salmonella Paratyphi OR enteric fever) AND (Bangladesh OR Nepal OR Pakistan) AND (blood culture) AND (incidence)”. Typhoid fever incidence from previous cohort studies and control groups of vaccine trials was 210–452 per 100 000 person-years in Karachi, Pakistan, 428–1062 per 100 000 in Lalitpur, Nepal, and 635–1135 in Dhaka, Bangladesh, with variation over time and by estimation method. Where reported, *Salmonella* Paratyphi A incidence was substantially lower. Most studies estimated incidence to be highest among children younger than 10 years.

**Added value of this study**

Using a hybrid surveillance approach combining both active clinical surveillance with health-care utilisation surveys, we report new, robust estimates of enteric fever incidence from population-based surveillance in Karachi, Pakistan; Kathmandu and Kavrepalanchok, Nepal; and Dhaka, Bangladesh. Adjusted incidences of typhoid per 100 000 person-years were 913 (95% CI 765–1095) in Dhaka, Bangladesh; 330 (230–480) in Kathmandu, Nepal; 268 (202–362) in Kavrepalanchok, Nepal; and 103 (85–126) and 176 (144–216) in the two surveillance sites for Karachi, Pakistan. The highest incidence was among children aged 2–4 years in both Bangladesh and Pakistan, and among individuals aged 5–25 years in Nepal. The adjusted incidence of hospitalisation of typhoid cases ranged from 249 (69–1191) per 100 000 person-years in Kathmandu, Nepal to 38 (28–57) per 100 000 person-years at Aga Khan University Hospital in Karachi, Pakistan. *Salmonella* Paratyphi A incidence per 100 000 person-years ranged from 1 (1–1) in one catchment area in Karachi to 81 (56–118) in Kathmandu and 128 (107–154) in Dhaka. These estimates represent the first population-based estimates for severe enteric fever, highlighting a substantial burden of hospitalisation for enteric fever in these communities. Our findings also document a high incidence of typhoid across all ages of children.

**Implications of all the available evidence**

The high incidence of typhoid and notable rates of hospitalisation in these study sites provides evidence needed to support the introduction of the typhoid conjugate vaccine in these settings, and the substantial burden among older children and young adults highlights the need for catch-up campaigns in addition to immunisation of young children.

In 2018, WHO recommended the use of the first typhoid conjugate vaccine (TCV) for children as young as 6 months in typhoid-endemic areas.^6^ Policy makers in typhoid-affected countries must now decide whether, and how, to introduce TCV into routine immunisation schedules, including the optimal age, target population, and delivery strategies. To make these decisions, timely and accurate information on enteric fever disease burden is required, including incidence and disease outcome data and local geographical and demographic distribution of typhoid.^7^, ^8^, ^9^, ^10^

Previous typhoid burden estimates were generated using active facility-based or passive augmented household-based studies.^11^, ^12^ Recent blood culture sentinel surveillance studies are generating important data on the burden of disease in select countries in Asia and sub-Saharan Africa.^13^ Yet for many countries in south Asia, there is limited understanding of the true burden of enteric fever. The Surveillance for Enteric Fever in Asia Project (SEAP), an ongoing blood culture surveillance study, was initiated in 2016 to describe the incidence, severity, and cost of enteric fever in sites in Bangladesh, Nepal, and Pakistan. SEAP was a two-phase study, with a retrospective component (phase 1) from sites in India, Bangladesh, Nepal, and Pakistan,^14^, ^15^ and 3 years of prospective surveillance (phase 2) in Bangladesh, Nepal, and Pakistan. SEAP phase 2 findings on cost of illness, antimicrobial resistance, patterns of health-care utilisation, severity of illness, blood culture use, and clinical, behavioural, and demographic factors of enteric fever were published in 2020.^16^ Findings from the prospective surveillance in India, the Surveillance for Enteric Fever in India project, will be published elsewhere. Here, we present the enteric fever incidence estimates from SEAP phase 2, with the aim of informing policy makers in disease prevention and control strategies.

## Methods

### Study design and participants

SEAP used a hybrid surveillance model, pairing facility-based surveillance with community health-care utilisation surveys to generate population-based enteric fever incidence estimates.^17^

We conducted prospective clinical surveillance from September, 2016, to September, 2019, at six tertiary hospitals: Dhaka Shishu Hospital and Shishu Shasthya Foundation Hospital in Dhaka, Bangladesh; Kathmandu Medical College and Teaching Hospital (KMC) in Kathmandu, and Dhulikhel Hospital, Kathmandu University Hospital (DH-KUH) in Kavrepalanchok District, Nepal; and Aga Khan University Hospital (AKUH), and Kharadar General Hospital (KGH) in Karachi, Pakistan. We also conducted surveillance within the surgical wards of Jinnah Postgraduate Medical Centre (JPMC) and the National Institute for Child Health (NICH) in Karachi. Hospital sites in Bangladesh were paediatric facilities serving patients aged 15 years or younger, whereas sites in Nepal and Pakistan served both children and adults. We also enrolled patients with blood culture-positive enteric fever from 15 laboratory network sites in all three countries (appendix p 2) to expand our recruitment reach to patients seeking care outside the participating study hospitals. The laboratory network sites were hospitals and clinics with laboratory capacity within the defined catchment areas that served both adult and paediatric inpatients and outpatients. Patients were therefore enrolled from a total of 23 health facilities. Study site selection (of the six tertiary hospitals) is described elsewhere.^16^, ^17^

Using address information from patients with blood culture-confirmed enteric fever identified from SEAP phase 1, we defined catchment areas around each study site hospital whereby more than 60% of the facility's cases were included.^18^ We then used census data or, in locations where a census was not available, a geographically based random sample to estimate the catchment area population size and the age distribution; the latter enabled us to capture changes due to migration during the study period.

All SEAP study site hospitals had a distinct catchment area, except for the two site hospitals in Bangladesh, which shared a catchment area. The catchment populations included an urban and periurban area of approximately 3·4 million people in Dhaka, Bangladesh; an urban, periurban, and rural catchment area of approximately 33 000 people in Kathmandu and Kavrepalanchok district, Nepal; and an urban and periurban area of approximately 3 million people, including slum settlements, in Karachi, Pakistan.

Study participants were individuals of all ages with febrile illness. Enrolment was offered to (1) outpatients (including emergency department patients) residing within the defined catchment area with 3 or more consecutive days of fever in the last 7 days; (2) inpatients with suspected or confirmed enteric fever; (3) patients with blood culture-confirmed enteric fever from the hospital laboratories not captured by inpatient or outpatient enrolment and cases from the laboratory network; and (4) surgical patients with non-traumatic ileal perforation regardless of microbial confirmation. At enrolment, interviewers asked questions on symptoms, medication history, and care sought for the current illness. Interviewers also asked questions on head of household education and household assets to every other patient enrolled in outpatient and inpatient wards, and to all patients with blood culture-confirmed enteric fever. All patients with blood culture-confirmed enteric fever were contacted by telephone 6 weeks following enrolment to ascertain self-reported hospitalisation after the enrolment visit.

We obtained written informed consent from all participants and the parents or guardians of participants younger than 18 years before blood collection and completion of the questionnaire. We obtained written assent from children aged 11–15 years in Bangladesh and from children aged 15–17 years in Nepal and Pakistan. Ethics review boards in the USA (Centers for Disease Control and Prevention and Stanford University Institutional Review Board), Bangladesh (Bangladesh Institute of Child Health Ethical Review Committee), Nepal (Nepal Health Research Council and Institutional Review Committee, DH-KUH, and KMC), and Pakistan (AKUH Ethics Review Committee and Pakistan National Bioethics Committee) approved the study forms and protocols.

### Laboratory methods and data collection

Whole blood was collected before treatment and incubated for up to 5 days using the BACTEC automated culture system (Becton Dickinson, Franklin Lakes, NJ, USA), or the BacTAlert 3D automated blood culture system (BioMérieux, Marcy-l’Étoile, France). Positive samples were subcultured onto MacConkey agar, chocolate agar, or sheep blood agar (or a combination of these), and species were confirmed using biochemical testing and O and H antisera (MAST ASSURE, Mast Group, Liverpool, UK), if available.^19^ Isolates obtained from laboratory network sites were confirmed at the study hospital laboratories using the same methods. All hospital laboratories were enrolled in WHO or College of American Pathologists external quality assurance programmes.

To estimate the proportion of individuals with a febrile illness in the catchment area compatible with enteric fever who sought care at the study facilities, interviewers administered a standard questionnaire to all households within randomly selected clusters of the defined catchment areas. The design and implementation of the health-care utilisation survey, including sample size calculations, are described elsewhere.^20^, ^21^

We defined disease severity by hospitalisation. Hospitalisation at the enrolment visit was documented in the medical charts, and hospitalisation during the 6-week follow-up period was patient-reported.

### Statistical analysis

We generated overall and age-specific typhoid and paratyphoid incidence estimates for each study site. As the Bangladesh sites shared a catchment area, the incidence rates are presented together. The total number of blood culture-confirmed cases was annualised according to the recruitment period for each facility. The annualised cases were then divided by the catchment area population size to calculate the incidence rate.

We calculated crude incidence rates among patients from within the hospital catchment areas with a positive blood culture enrolled from outpatient, inpatient, and surgical wards, as well as from hospital laboratories and laboratory networks. In Nepal and Pakistan, patients of any age were included. In Bangladesh, only children aged 15 years or younger were included in the calculations, as the study site hospitals were paediatric facilities.

We calculated adjusted incidence among patients with blood culture-confirmed enteric fever from within the catchment areas enrolled from the outpatient, inpatient, and surgical wards only. Patients recruited from laboratories were excluded from the adjusted incidence estimates (appendix pp 3–4) as participants with febrile illness were not systematically enrolled and offered blood cultures at those sites, precluding adjustment for participation. The unadjusted incidence, different from the crude incidence, describes the rate of age-eligible case patients recruited from outpatient, inpatient, and surgical wards before adjustments were applied.

The adjusted incidence estimates included adjustments for (1) the sensitivity of blood culture (59%);^22^ (2) the proportion of eligible individuals who consented and provided blood; (3) the probability of seeking care at a study site facility for individuals with suspected enteric fever (data from the health-care utilisation survey); and (4) the influence of wealth and education on care-seeking at a study site (differential care-seeking). For further details on adjustments see the appendix (pp 1, 3–4).

The incidence of hospitalisation was calculated among outpatients, inpatients, and surgical ward patients who were hospitalised at the enrolment visit or were reportedly hospitalised in the 6 weeks after enrolment. The adjustments included (1) the sensitivity of blood culture; (2) the proportion of patients admitted to hospital who consented and provided blood; (3) the probability of being hospitalised for fever (data from the health-care utilisation survey); and (4) the influence of wealth and education on being hospitalised (differential care-seeking for hospitalisation).

For each adjustment, we used a Monte Carlo approach to create distributions for each adjustment probability based on 1 million simulations and derived the median, 2·5th, and 97·5th percentiles. Age-specific adjustment factors for the adjusted incidence calculations are detailed in the appendix (pp 5–7).

All analyses were performed in R (version 4.1.2).

### Role of the funding source

The funder of the study had no role in study design, data collection, data analysis, data interpretation, or writing of the report.

## Results

**Figure 1:**
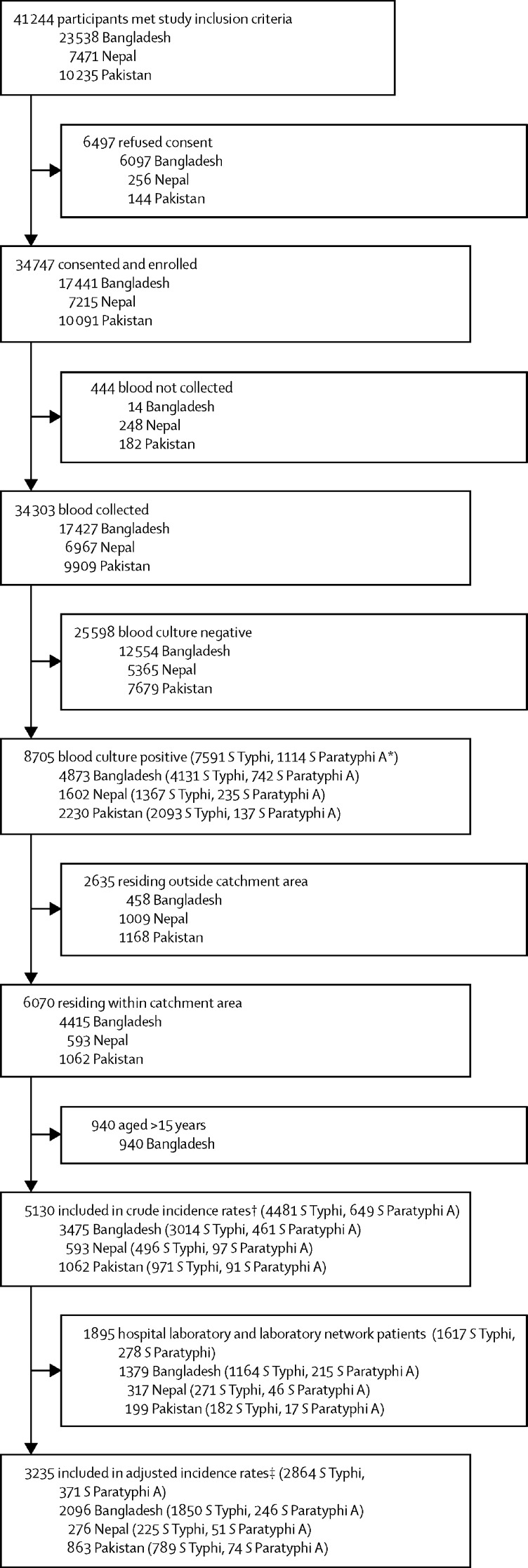
Recruitment, eligibility, study consent, and laboratory culture positivity *Two detected cases of *S* Paratyphi B were excluded. †Population for the crude incidence rates includes age-eligible patients from within the catchment area recruited from outpatient departments, inpatient departments, surgical wards, hospital laboratories, and laboratory network sites. ‡Population for the adjusted incidence rates includes age-eligible patients from within the catchment area recruited from outpatient departments, inpatient departments, and surgical wards.

Across all surveillance sites, 41 244 individuals met the study inclusion criteria during the study period, 34 747 (84%) of whom consented and were enrolled: 17 441 (74%) of 23 538 in Bangladesh, 7215 (97%) of 7471 in Nepal, and 10 091 (99%) of 10 235 in Pakistan (figure 1). Blood cultures were performed for 34 303 (99%) of 34 737 enrolled patients, with 8705 (26%) positive for enteric fever (4873 [28%] of 17 427 in Bangladesh, 1602 [23%] of 6967 in Nepal, and 2230 [23%] of 9909 in Pakistan). Most typhoidal *Salmonella* isolates across countries were *S* Typhi: 4131 (85%) of 4873 in Bangladesh, 1367 (85%) of 1602 in Nepal, and 2093 (94%) of 2230 in Pakistan (figure 1). Of a total of 1116 *S* Paratyphi isolates, 1114 (>99%) were Paratyphi A. Two cases of Paratyphi B were detected (0·2%) and were excluded from the analyses. No cases of Paratyphi C were detected.

19 868 (57%) of the enrolled participants were male and 14 879 (43%) were female. Most were recruited from outpatient facilities (20 899 [60%]), whereas 248 (1%) entered the study through surgical wards. The median age of participants was 6 years (IQR 2–17), and participants from Nepal were generally older than those from Bangladesh and Pakistan (table 1). Although the tertiary hospitals in Bangladesh were paediatric facilities, 1215 patients older than 15 years were enrolled from laboratory network sites.

**Table 1:** Demographic characteristics of patients enrolled (consented)

		**Bangladesh (n=17 441)**	**Nepal (n=7215)**	**Pakistan (n=10 091)**	**Overall (n=34 747)**
**Overall enrolment**					
Age, years		4 (2–7)	20 (8–31)	8 (3–22)	6 (2–17)
	<2 years	3751 (22%)	363 (5%)	1645 (16%)	5759 (17%)
	2–4 years	5708 (33%)	780 (11%)	1982 (20%)	8470 (24%)
	5–15 years	6757 (39%)	1408 (20%)	2916 (29%)	11 081 (32%)
	16–25 years	733 (4%)	2086 (29%)	1234 (12%)	4053 (12%)
	>25 years	482 (3%)	2578 (36%)	2314 (23%)	5384 (15%)
	6 months–15 years	16 071 (92%)	2655 (37%)	6440 (64%)	25 166 (72%)
Sex					
	Male	9912 (57%)	4204 (58%)	5752 (57%)	19 868 (57%)
	Female	7529 (43%)	3007 (42%)	4339 (43%)	14 879 (43%)
Study recruitment location					
	Outpatient department	10 991 (63%)	5047 (70%)	4861 (48%)	20 899 (60%)
	Inpatient department	3760 (22%)	866 (12%)	4209 (42%)	8835 (25%)
	Surgical wards	2 (<1%)	5 (<1%)	241 (2%)	248 (1%)
	Hospital laboratory	847 (5%)	229 (3%)	199 (2%)	1275 (4%)
	Laboratory network site	1841 (11%)	1068 (15%)	581 (6%)	3490 (10%)
**Laboratory-confirmed enteric fever cases**					
Total		4873 (28%)	1602 (22%)	2230 (22%)	8705 (25%)
Age, years		6 (3–14)	20 (15–25)	6 (3–13)	8 (4–19)
	<2 years	449 (9%)	18 (1%)	305 (14%)	772 (9%)
	2–4 years	1314 (27%)	46 (3%)	622 (28%)	1982 (23%)
	5–15 years	1938 (40%)	313 (20%)	818 (37%)	3069 (35%)
	16–25 years	688 (14%)	818 (51%)	288 (13%)	1794 (21%)
	>25 years	484 (10%)	407 (25%)	197 (9%)	1088 (12%)
	6 months–15 years	3733 (77%)	415 (26%)	479 (21%)	5899 (68%)
Sex					
	Male	2754 (57%)	942 (59%)	1286 (58%)	4982 (57%)
	Female	2119 (43%)	660 (41%)	944 (42%)	3723 (43%)
Study recruitment location					
	Outpatient department	1453 (30%)	266 (17%)	694 (31%)	2413 (28%)
	Inpatient department	732 (15%)	38 (2%)	748 (34%)	1518 (17%)
	Surgical wards	0 (0%)	1 (<1%)	8 (<1%)	9 (<1%)
	Hospital laboratory	847 (17%)	229 (14%)	199 (9%)	1275 (15%)
	Laboratory network site	1841 (38%)	1068 (67%)	581 (26%)	3490 (40%)
**Hospitalised laboratory-confirmed enteric fever cases**					
Total		1295 (27%)	455 (28%)	1054 (47%)	2804 (32%)
Age, years		5 (3–10)	19 (14–24)	6 (3–13)	7 (3–16)
	<2 years	169 (13%)	7 (2%)	134 (13%)	310 (11%)
	2–4 years	419 (32%)	10 (2%)	288 (27%)	717 (26%)
	5–15 years	501 (39%)	103 (23%)	391 (37%)	995 (35%)
	16–25 years	126 (10%)	230 (51%)	145 (14%)	501 (18%)
	>25 years	80 (6%)	105 (23)	96 (9%)	281 (10)
	6 months–15 years	1091 (84%)	138 (30%)	829 (79%)	2058 (73%)
Sex					
	Male	733 (57%)	238 (52%)	618 (59%)	1589 (57%)
	Female	562 (43%)	217 (48%	436 (41%)	1215 (43%)
Study recruitment location					
	Outpatient department	125 (10%)	53 (12%)	114 (11%)	292 (10%)
	Inpatient department	732 (57%)	38 (8%)	748 (71%)	1518 (54%)
	Surgical wards	0 (0%)	1 (<1%)	8 (1%)	9 (<1%)
	Hospital laboratory	83 (6%)	58 (13%)	21 (2%)	162 (6%)
	Laboratory network site	355 (27%)	305 (67%)	163 (15%)	823 (29%)

Data are median (IQR) or n (%). For the data beneath each row marked “Total”, the denominators for the percentages are the numbers of patients listed in the respective “Total” rows.

Among all patients with laboratory-confirmed typhoid, the median age was 7 years (IQR 3–18), while the median age of patients with laboratory-confirmed paratyphoid was 12 years (5–23). Overall, 2804 (32%) patients with laboratory-confirmed enteric fever were hospitalised at the enrolment visit, with the highest percentage in Pakistan (Bangladesh 27%, Nepal 28%, and Pakistan 47%; table 1). Across countries, the majority of hospitalised patients were male (male 1589 [57%], female 1215 [43%]), and the median age was 7 years (3–16; table 1).

Among the 34 747 patients enrolled, 33 488 (96%) resided within the catchment areas and met the age criteria and were included in the crude incidence analysis; 31 593 (91%) were recruited from outpatient, inpatient, and surgical wards and were included in the adjusted incidence analysis. None of the patients who were blood culture-positive from JPMC and NICH resided within the catchment areas. Overall, 2864 typhoid cases and 371 paratyphoid cases were included in the incidence rate calculations.

The crude incidence rates of enteric fever, expressed as cases per 100 000 person-years, varied by and within countries. The crude incidence rates of typhoid ranged from 103 (95% CI 97–109) per 100 000 person-years in Bangladesh to 12 (10–14) per 100 000 person-years at AKUH in Pakistan (table 2). In Bangladesh, the crude typhoid incidence was highest among children aged 2–4 years (217 [195–239] per 100 000 person-years), whereas in both sites in Pakistan, the incidence was highest among children younger than 2 years. In both sites in Nepal, crude typhoid incidence peaked in patients aged 16–25 years. The crude rates of paratyphoid were 16 (13–18) per 100 000 person-years in Bangladesh; 7 (3–16) per 100 000 person-years at DH-KUH and 6 (4–9) per 100 000 person-years at KMC, in Nepal; and 3 (2–4) per 100 000 person-years at AKUH and 0 (0–1) per 100 000 person-years at KGH, in Pakistan, following similar age trends as the crude incidence of typhoid (table 2).

**Table 2:** Crude and fully adjusted incidence rates of laboratory-confirmed *Salmonella* yphi and *Salmonella* Paratyphi A and incidence of hospitalisation per 100 000 person-years, by age group

	**Incidence of enteric fever**				**Incidence of hospitalisation due to enteric fever**			
	*Salmonella* Typhi		*Salmonella* Paratyphi A		*Salmonella* Typhi		*Salmonella* Paratyphi A	
	Crude incidence per 100 000 person-years	Fully adjusted incidence per 100 000 person-years	Crude incidence per 100 000 person-years	Fully adjusted incidence per 100 000 person-years	Crude incidence per 100 000 person-years	Fully adjusted incidence per 100 000 person-years	Crude incidence per 100 000 person-years	Fully adjusted incidence per 100 000 person-years
**Bangladesh: Dhaka Shishu Hospital and Shishu Shasthya Foundation Hospital**								
<2 years	160 (133–191)	780 (638–968)	22 (13–35)	81 (66–101)	59 (43–79)	374 (300–507)	4 (1–11)	38 (30–51)
2–4 years	217 (195–239)	1491 (1244–1804)	31 (23–41)	188 (157–228)	69 (57–82)	557 (448–755)	8 (4–13)	69 (56–94)
5–15 years	93 (86–101)	1302 (1038–1642)	15 (12–19)	208 (165–262)	24 (20–28)	200 (161–271)	3 (2–5)	30 (24–41)
Overall	103 (97–109)	913 (765–1095)	16 (13–18)	128 (107–154)	30 (27–33)	242 (195–328)	3 (2–5)	33 (26–44)
**Nepal: Dhulikhel Hospital**								
<2 years	37 (1–311)	156 (100–259)	NA*	NA*	NA*	NA*	NA*	NA*
2–4 years	19 (1–164)	159 (102–262)	10 (0–109)	NA*	10 (0–109)	53 (36–151)	NA*	NA*
5–15 years	60 (28–116)	570 (310–1124)	9 (0–38)	114 (62–224)	31 (11–79)	123 (85–348)	2 (0–25)	25 (17–70)
16–25 years	67 (36–123)	406 (219–820)	17 (4–51)	117 (65–233)	13 (1–42)	103 (70–290)	2 (0–22)	32 (22–92)
>25 years	18 (9–36)	172 (119–256)	3 (0–12)	9 (6–14)	4 (1–15)	38 (26–106)	NA*	NA*
Overall	36 (24–51)	268 (202–362)	7 (3–16)	46 (34–62)	10 (5–20)	67 (46–189)	1 (0–7)	12 (8–34)
**Nepal: Kathmandu Medical College**								
<2 years	8 (0–71)	251 (120–613)	9 (0–71)	NA*	8 (0–71)	178 (48–850)	NA*	NA*
2–4 years	28 (8–72)	227 (102–522)	9 (0–39)	227 (103–521)	12 (2–51)	140 (38–672)	NA*	NA*
5–15 years	46 (31–65)	423 (228–826)	7 (2–15)	66 (35–153)	16 (8–29)	398 (107–1890)	1 (0–8)	60 (16–287)
16–25 years	68 (53–84)	653 (313–1440)	12 (7–20)	169 (79–381)	19 (11–28)	504 (135–2394)	2 (0–6)	131 (35–624)
>25 years	11 (7–16)	130 (76–230)	3 (1–6)	34 (20–61)	2 (1–5)	96 (26–458)	1 (0–3)	25 (7–120)
Overall	31 (26–37)	330 (230–480)	6 (4–9)	81 (56–118)	9 (6–12)	249 (67–1191)	1 (0–3)	61 (16–291)
**Pakistan: Aga Khan University Hospital**								
<2 years	43 (24–74)	282 (191–449)	1 (0–12)	10 (6–18)	20 (7–41)	138 (102–208)	1 (0–12)	4 (3–6)
2–4 years	36 (23–54)	371 (265–534)	4 (0–10)	38 (26–56)	19 (10–32)	116 (86–175)	1 (0–8)	9 (7–14)
5–15 years	25 (19–32)	280 (201–397)	3 (2–7)	42 (30–60)	11 (7–16)	71 (52–107)	1 (0–3)	9 (7–14)
16–25 years	11 (7–17)	152 (103–230)	4 (2–8)	60 (41–92)	5 (2–9)	36 (26–54)	1 (0–2)	14 (10–21)
>25 years	3 (2–5)	22 (18–28)	2 (1–3)	10 (8–13)	1 (0–2)	10 (8–16)	0 (0–1)	4 (3–6)
Overall	12 (10–14)	103 (85–126)	3 (2–4)	23 (19–29)	6 (4–7)	38 (28–57)	1 (0–1)	8 (6–11)
**Pakistan: Kharadar General Hospital**								
<2 years	175 (122–244)	628 (470–889)	4 (0–28)	8 (6–12)	40 (17–79)	422 (307–708)	NA*	NA*
2–4 years	146 (113–185)	901 (662–1269)	1 (0–8)	6 (4–8)	36 (22–59)	394 (287–664)	NA*	NA*
5–15 years	29 (21–38)	344 (249–483)	0 (0–2)	NA*	5 (2–10)	88 (64–147)	NA*	NA*
16–25 years	3 (1–8)	36 (24–57)	NA*	NA*	1 (0–4)	10 (8–18)	NA*	NA*
>25 years	1 (0–3)	8 (6–10)	NA*	NA*	0 (0–2)	3 (2–5)	NA*	NA*
Overall	24 (21–28)	176 (144–216)	0 (0–1)	1 (1–1)	5 (3–7)	64 (46–107)	NA*	NA*

Crude incidence includes patients from within the catchment area recruited from outpatient departments, inpatient departments, surgical wards, hospital laboratories, and laboratory network sites. Fully adjusted incidence includes patients from within the catchment area recruited from outpatient departments, inpatient departments, and surgical wards only. NA=not applicable.

* No observed cases and therefore insufficient data to estimate incidence.

**Figure 2:**
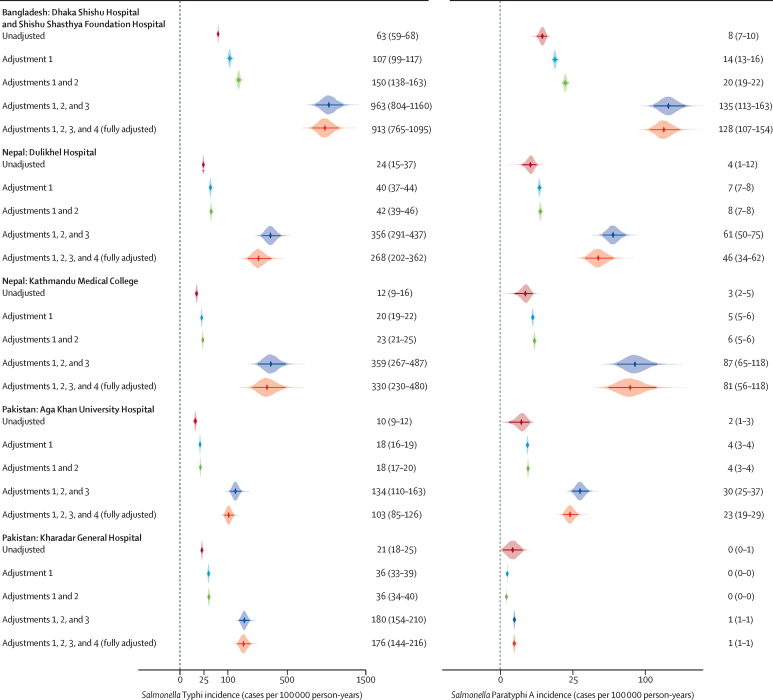
Unadjusted and adjusted incidence rates of laboratory-confirmed typhoid and paratyphoid cases per 100 000 person-years, by adjustment factor and by study site Adjustments are as follows: adjustment 1=culture sensitivity; adjustment 2=study consent; adjustment 3=care-seeking; adjustment 4=differential care-seeking. The points indicate the median estimate and shaded areas indicate the density. The numbers to the right of each graph indicate the incidence estimate and the 95% confidence interval for each adjustment.

The ranking of the fully adjusted rates of typhoid by site followed that of the crude incidence. The adjusted typhoid incidence observed in Bangladesh was 913 (95% CI 765–1095) per 100 000 person-years. In Nepal, the observed incidence at DH-KUH was 268 (202–362) per 100 000 person-years and at KMC was 330 (230–480) per 100 000 person-years; and in Pakistan, it was 103 (85–126) per 100 000 person-years at AKUH and 176 (144–216) per 100 000 person-years at KGH (table 2, figure 2).

**Figure 3:**
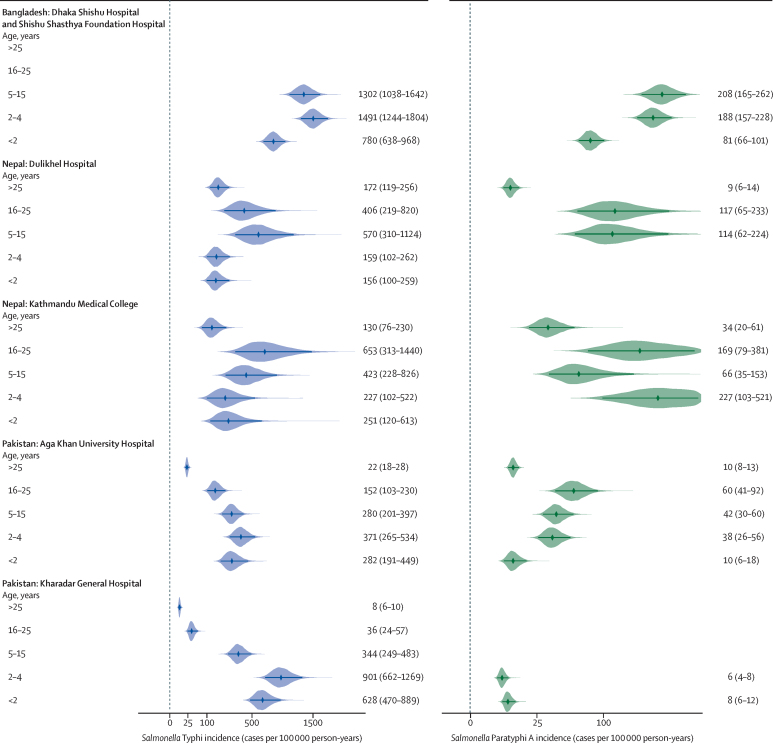
Adjusted incidence estimates of typhoid and paratyphoid cases per 100 000 person-years, by age group and by study site The points indicate the median estimate and shaded areas indicate the density. The numbers to the right of each graph indicate the incidence estimate and the 95% confidence interval for each age group. There are no data plotted for *Salmonella* Typhi and Paratyphi A incidence among people aged 16–25 years and >25 years for the Bangladesh sites because these were paediatric facilities. There are no data plotted for *Salmonella* Paratyphi A incidence among people aged <2 years and 2–4 years in Dulikhel Hospital (Nepal), aged <2 years in Kathmandu Medical College (Nepal), and aged 5–15 years, 16–25 years, and >25 years in Kharadar General Hospital (Pakistan), because there were no observed cases for these age groups and therefore insufficient data to estimate incidence.

A high adjusted incidence of typhoid in children younger than 2 years was observed in all study sites (table 2, figure 3). The highest incidence was among children aged 2–4 years in both Bangladesh and Pakistan, and among individuals aged 5–25 years in Nepal. Estimated incidence decreased with age in the older age groups in Bangladesh and Pakistan, whereas it remained high in individuals aged 5–25 years in Nepal (figure 3). All incidence adjustments by age are detailed in the appendix (pp 5–7).

Although lower than the rates of typhoid, the fully adjusted incidence rates of paratyphoid followed the same pattern, with the highest rates in Bangladesh and the lowest in Pakistan (table 2, figure 2). In Bangladesh, however, the highest incidence of paratyphoid was among children aged 5–15 years (208 [95% CI 165–262] per 100 000 person-years; table 2, figure 3). At DH-KUH in Nepal and AKUH in Pakistan, the highest incidence was among participants aged 16–25 years (117 [65–233] per 100 000 person-years at DH-KUH and 60 [41–92] per 100 000 person-years at AKUH). At KMC in Nepal, the highest adjusted incidence was among children aged 2–4 years (227 [103–521] per 100 000 person-years). There were no observed cases of paratyphoid among participants older than 4 years at KGH in Pakistan (figure 3).

The adjusted incidence of hospitalisation of patients with typhoid ranged from 249 (95% CI 67–1191) per 100 000 person-years at KMC in Nepal to 38 (28–57) per 100 000 person-years at AKUH in Pakistan. Incidence of hospitalisation was highest among children aged 2–4 years in Bangladesh; among individuals aged 5–15 years in DH-KUH and 16–25 years in KMC, in Nepal; and among children younger than 2 years in both sites in Pakistan. The highest incidence of hospitalisation of patients with paratyphoid was among the same age groups as those for typhoid in Bangladesh and KMC in Nepal. However, the adjusted incidence of hospitalisation at DH-KUH in Nepal and AKUH in Pakistan was highest among individuals aged 16–25 years. No patients with paratyphoid were hospitalised at KGH in Pakistan (table 2).

## Discussion

We conducted prospective, population-based enteric fever surveillance for 3 years to generate incidence data on typhoid and paratyphoid in mostly urban and periurban communities within Bangladesh, Nepal, and Pakistan. Our incidence estimates—crude, unadjusted, and adjusted rates—show a substantial burden of enteric fever in all participating sites. Crude incidence rates are a minimal estimate for enteric fever incidence and are highly sensitive to the coverage of surveillance facilities, which varied across catchment areas. Adjusted rates varied by country, between different communities in a country (eg, Kathmandu *vs* Kavrepalanchok, Nepal), and by hospitals in the same city (eg, AKUH *vs* KGH in Karachi, Pakistan). However, regardless of population characteristics (eg, highly populated urban areas or lower density periurban communities), all fully adjusted incidence estimates exceeded the 100 per 100 000 person-years threshold used to define “high burden” settings for enteric fever, with some surpassing the “very high” incidence threshold (≥500 cases per 100 000 person-years).^11^, ^22^, ^23^ In addition to high overall incidence rates, we observed a high adjusted incidence of severe disease among case patients. The high rates of hospitalisation indicate considerable morbidity and economic impact, highlighting the need for control measures, including the implementation of TCV.^24^, ^25^, ^26^

Our findings are consistent with previous studies identifying typhoid in young children;^27^, ^28^, ^29^ our age-specific incidence rates showed a high burden of disease in children younger than 5 years, including those younger than 2 years. These data emphasise the need to start TCV immunisation as early as age 6 months, as recommended by WHO.^6^ However, in Nepal, the adjusted incidence of disease was higher among individuals 5 years and older, highlighting the importance of understanding local epidemiology for a tailored and more effective vaccination strategy.

The hybrid surveillance approach used to estimate incidence has several strengths. It allowed us to surveil a large population and use systematically collected data to adjust crude estimates to generate empirically based incidence estimates.^20^ This hybrid framework captures a full spectrum of disease, unlike cohort studies where intensive, early testing and close follow-up might bias towards milder cases. In our study, the large number of confirmed cases improved the precision of our estimates and permitted reliable, robust stratified analyses. Additionally, we selected diverse surveillance sites and investigated demographic determinants of care-seeking through household surveys, adjusting for factors associated with care-seeking and typhoid risk in the analyses to reduce bias in the estimates.^30^

There were limitations to this study. Although we generated robust catchment-area-specific incidence estimates, our estimates might not be generalisable to other areas outside of our surveillance sites, or to similar areas using different methods. A limitation to our approach was the reliance on self-reporting for medical history, hospitalisation after the enrolment visit, and socioeconomic information. Although there is the potential for recall bias in the medical history, the likelihood of imperfect recall is low for reported hospitalisations in the 6 weeks following enrolment given the short timeframe and no obvious incentive for participants to either exaggerate or minimise their symptoms. To mitigate misreporting of socioeconomic information, we used questions related to head of household education and household assets, which are more reliable and less sensitive than household income-related questions. Our study locations were selected on the basis of evidence of enteric fever^18^ and the capacity for blood culture-based surveillance, which might result in higher incidence compared with rural, less densely populated areas. More assessments in rural areas could help inform the use of nationwide TCV roll-out versus targeted vaccine campaigns. To further explore the burden of disease in other areas, an ongoing serosurveillance study was implemented in 2019 in the SEAP sites and in rural areas of each country. Serosurveillance could have the further advantage of measuring the true force of infection in a population, including subclinical infections that are missed by clinical surveillance. Similarly, we might have missed cases of enteric fever at our study sites due to the sensitivity of blood culture, which is even lower for patients with previous antibiotic use.^22^ Data on previous antibiotic use from within the SEAP sites are published elsewhere*.*^31^ To address this limitation, we included the estimated sensitivity of blood culture (59%) in our incidence adjustments. Lastly, we annualised incidence rates and therefore did not capture seasonality of typhoid transmission. However, we do not expect that seasonal variability in transmission to influence TCV introduction decisions.

The largest differences between the crude and adjusted incidence estimates were generated by the adjustment factor for health-care-seeking behaviours. This adjustment rests on the assumption that individuals with 3 days or more of fever in the population have the same rate of enteric fever infection as those who sought care. Although we showed that both care-seeking and blood culture positivity varied by household education and wealth status, when accounting for these variations, the final adjusted estimates changed only marginally compared with the estimates adjusted for health-care-seeking alone. It is possible that there are residual differences in health-care-seeking behaviour that we could not measure, and for which we could not adjust. Moreover, it is recognised that most patients with febrile illness in south Asia do not have blood culture performed,^32^ so a large adjustment in incidence for care-seeking is expected.

Accurate, context-specific burden data on enteric fever, such as those generated by SEAP, might inform TCV introduction and the implementation of other enteric fever prevention and control measures. Additionally, population-based surveillance studies can serve as a platform to measure the impact of vaccine introduction. In the long term, surveillance can identify waning protection and the need for revaccination, describe the impact of vaccination on antimicrobial resistance, and measure potential serotype replacement by *S* Paratyphi A.

In conclusion, the high burden of typhoid in the SEAP study sites suggests that these communities would benefit from TCV introduction. Early data from SEAP contributed to the decision to introduce TCV in Pakistan, where the Government launched the first nationwide TCV introduction in 2019. In addition to data on typhoid incidence, it is also important to monitor the burden of paratyphoid as these data will also help inform the introduction of upcoming bivalent *S* Typhi and *S* Paratyphi A vaccines. Continued surveillance provides insight into how factors such as increased urbanisation, climate change, decreasing per capita water availability, and rising drug resistance affect the epidemiology and burden of enteric fever.

## Data sharing

De-identified study data can be shared upon request with investigators who provide a methodologically sound proposed use of the data, which will be determined by the principle investigators, including the corresponding author. Data requests should be made by contacting the corresponding author.

## Declaration of interests

We declare no competing interests.
