# Supplementary material for: Incidence of typhoid and paratyphoid fever in Bangladesh, Nepal, and Pakistan: results of the Surveillance for Enteric Fever in Asia Project
Source: Lancet Glob Health. 2022 Jun 14;10(7):e978–88. doi: 10.1016/S2214-109X(22)00119-X (PMC9210262; doi:10.1016/S2214-109X(22)00119-X)
Supplement: Supplementary appendix [file mmc1.pdf]

THE LANCET
Global Health

Supplementary appendix

This appendix formed part of the original submission and has been peer reviewed. We post it as supplied by the authors.

Supplement to: Garrett DO, Longley AT, Aiemjoy K, et al. Incidence of typhoid and paratyphoid fever in Bangladesh, Nepal, and Pakistan: results of the Surveillance for Enteric Fever in Asia Project. *Lancet Glob Health* 2022; **10:** e978–88.

Supplementary Methods

The sensitivity of blood culture was estimated to be 0·59 (95% confidence interval [CI]: 0·54–0·64) based on a recent meta-analysis by Antillon *et al*.[i]

The adjustment for consent and blood culture utilization was estimated by dividing the number of eligible persons who consented and provided a blood sample by the number of participants who were eligible for study enrollment at the SEAP hospitals in each age strata. These probabilities were estimated separately for inpatient and outpatient enrollment (Supplementary Table 2).

The adjustment for care-seeking was estimated from the health care utilization survey, using the probability of individuals with suspected enteric fever (defined as ≥3 consecutive days of reported fever in a 7-day period) seeking care or being hospitalized with febrile illness at a study site. Generalized estimating equations were used to account for clustering by geographic sampling unit.[ii] Age-specific probabilities were calculated for outpatient care-seeking, and overall probabilities (not age-specific) were used for inpatient care-seeking (Supplementary Table 2).

For the last adjustment, differential care-seeking, we created a wealth index using a principal components analysis[iii] incorporating the following assets: electricity and ownership of radio, television, landline telephone, mobile phone, computer, watch, bicycle, motorcycle, car, and bank account for study participants in both the health care utilization survey and the clinical surveillance studies. We then used inverse probability weighting approach to account for differences in care-seeking according to wealth and household education, using male head of household education as the indicator variable. We generated propensity scores based on the probability of being blood culture-positive for enteric fever according to education level of the male head of household and household wealth index in the clinical data. We then applied these scores as inverse weights to the health care utilization data to generate estimates that account for the care-seeking behavior adjusted for education and wealth. In Nepal, we combined the <2 and 2-4 differential care seeking adjustment because there were too few households in each age strata to calculate stratified adjustments (Supplementary Table 2).

Supplementary Tables

Supplementary Table 1. Laboratory network sites conducting blood culture surveillance for the Surveillance for Enteric Fever in Asia Project (SEAP)— Bangladesh, Nepal, and Pakistan, 2016-2019

Country	Laboratory Network Sites
Bangladesh	Popular Diagnostic Center - Dhanmondi
	Popular Diagnostic Center - Mirpur
	Popular Diagnostic Center - Shamoly
Nepal	Alka Hospital
	Nepal Medical College
	Kathmandu Model Hospital
	Bir Hospital
	Helping Hands Clinic
	Nepal Police Hospital
	Kanti Children Hospital
	Nepal Army Hospital
Pakistan	AKU Secondary Hospital Garden Collection Unit
	AKU Secondary Hospital Kharadar Collection Unit
	AKUH Main Laboratory Collection Unit
	KGH Hospital Lab / Lab Network

Supplementary Table 2. Outpatient and inpatient adjustment factors used to estimate incidence of typhoid and paratyphoid fevers by age and study site, Surveillance for Enteric Fever in Asia Project (SEAP) — Bangladesh, Nepal, and Pakistan, 2016-2019

	**Adjustment 1: Blood Culture Sensitivity**	**Adjustment 2: Study Consent**	**Adjustment 3: Care-Seeking**	**Adjustment 4: Differential Care-Seeking**
		**Outpatient**		
**Bangladesh**				
<2 years	0·59	0.666 DSH; 0.520 SSF	0.221	0.234
2 - 4 years	0·59	0.738 DSH; 0.556 SSF	0.188	0.195
5 - 15 years	0·59	0.751 DSH; 0.575 SSF	0.072	0.078
Total	0·59	0.723 DSH; 0.553 SSF	0.128	0.135
**Dhulikhel Hospital, Kathmandu University Hospital**				
<2 years	0·59	0.934	0.143	0.213
2 - 4 years	0·59	0.972	0.189	0.213
5 - 15 years	0·59	0.964	0.123	0.137
16-25 years	0·59	0.950	0.128	0.155
>25 years	0·59	0.944	0.087	0.138
Total	0·59	0.953	0.118	0.156
**Kathmandu Medical College and Teaching Hospital**				
<2 years	0·59	0.878	0.060	0.056
2 - 4 years	0·59	0.925	0.067	0.056
5 - 15 years	0·59	0.950	0.085	0.087
16-25 years	0·59	0.897	0.047	0.070
>25 years	0·59	0.904	0.057	0.067
Total	0·59	0.911	0.061	0.069
**Aga Khan University Hospital**				
<2 years	0·59	0.950	0.141	0.182
2 - 4 years	0·59	0.927	0.092	0.118
5 - 15 years	0·59	0.945	0.066	0.095
16-25 years	0·59	0.958	0.076	0.098
>25 years	0·59	0.969	0.147	0.191
Total	0·59	0.954	0.110	0.144
**Kharadar General Hospital**				
<2 years	0·59	0.975	0.378	0.368
2 - 4 years	0·59	0.976	0.201	0.224
5 - 15 years	0·59	0.977	0.131	0.133
16-25 years	0·59	0.959	0.137	0.140
>25 years	0·59	0.980	0.191	0.193
Total	0·59	0.975	0.184	0.187

**Inpatient**				
**Bangladesh**				
<2 years	0·59	0.989 DSH; 0.960 SSF	0.613	0.619
2 - 4 years	0·59	0.988 DSH; 0.984 SSF	0.613	0.619
5 - 15 years	0·59	0.991 DSH; 0.967 SSF	0.613	0.619
Total	0·59	0.989 DSH; 0.972 SSF	0.613	0.619
**Dhulikhel Hospital, Kathmandu University Hospital**				
<2 years	0·59	NA	0.350	0.636
2 - 4 years	0·59	1.000	0.350	0.636
5 - 15 years	0·59	1.000	0.350	0.636
16-25 years	0·59	0.933	0.350	0.636
>25 years	0·59	1.000	0.350	0.636
Total	0·59	0.985	0.350	0.636
**Kathmandu Medical College and Teaching Hospital**				
<2 years	0·59	0.889	0.167	0.091
2 - 4 years	0·59	0.867	0.167	0.091
5 - 15 years	0·59	0.840	0.167	0.091
16-25 years	0·59	0.725	0.167	0.091
>25 years	0·59	0.685	0.167	0.091
Total	0·59	0.755	0.167	0.091
**Aga Khan University Hospital**				
<2 years	0·59	1.000	0.410	0.483
2 - 4 years	0·59	1.000	0.410	0.483
5 - 15 years	0·59	0.995	0.410	0.483
16-25 years	0·59	1.000	0.410	0.483
>25 years	0·59	0.994	0.410	0.483
Total	0·59	0.997	0.410	0.483
**Kharadar General Hospital**				
<2 years	0·59	0.990	0.488	0.572
2 - 4 years	0·59	1.000	0.488	0.572
5 - 15 years	0·59	0.974	0.488	0.572
16-25 years	0·59	1.000	0.488	0.572
>25 years	0·59	0.989	0.488	0.572
Total	0·59	0.989	0.488	0.572

Supplementary Table 3. Typhoid and Paratyphoid incidence estimates by age group, study site, and adjustment factor, Surveillance for Enteric Fever in Asia Project (SEAP) — Bangladesh, Nepal, and Pakistan, 2016-2019

								Incidence Estimates by Adjustment Factor n (95% CI)			
	Population	Inpatient cases per Anum	Outpatient cases per Anum	Total Cases per Anum[1]	Crude Incidence[1] (95% CI)	Cases per Anum[2]	Unadjusted Incidence[2] (95% CI)	Adjustment 1: Blood Culture Sensitivity	Adjustments 1 and 2: Blood Culture Sensitivity + Study Consent	Adjustments 1, 2, and 3: Blood Culture Sensitivity + Study Consent + Care-seeking	Adjustments 1, 2, 3, and 4: Blood Culture Sensitivity + Study Consent + Care-seeking+ Differential Care-Seeking
**Typhi**											
**Bangladesh**											
<2 years	77,958	35	41	125	160 (133 - 191)	77	98 (78 - 123)	166 (153 - 182)	231 (213 - 252)	820 (675 - 1008)	780 (638 - 968)
2 - 4 years	173,878	80	175	376	217 (195 - 239)	256	147 (130 - 166)	249 (230 - 272)	344 (317 - 376)	1542 (1299 - 1842)	1491 (1244 - 1804)
5 - 15 years	588,070	85	229	549	93 (86 - 101)	314	53 (48 - 60)	90 (83 - 99)	124 (114 - 135)	1412 (1129 - 1773)	1302 (1038 - 1642)
Total	1,019,847	201	445	1050	103 (97 - 109)	646	63 (59 - 68)	107 (99 - 117)	150 (138 - 163)	963 (804 - 1160)	913 (765 - 1095)
**Dhulikhel Hospital, Kathmandu University Hospital**											
<2 years	1,789	0	0	1	37 (1 - 311)	0	18 (0 - 206)	31 (29 - 34)	33 (31 - 36)	234 (141 - 406)	156 (100 - 259)
2 - 4 years	3,392	0	1	1	19 (1 - 164)	1	19 (1 - 164)	33 (30 - 36)	34 (31 - 37)	179 (130 - 252)	159 (102 - 262)
5 - 15 years	14,746	0	7	9	60 (28 - 116)	7	45 (19 - 98)	76 (70 - 82)	78 (72 - 86)	638 (455 - 910)	570 (310 - 1124)
16-25 years	17,101	0	6	12	67 (36 - 123)	6	37 (13 - 76)	62 (57 - 68)	65 (60 - 71)	494 (327 - 768)	406 (219 - 820)
>25 years	46,967	0	6	9	18 (9 - 36)	6	13 (5 - 28)	22 (21 - 25)	24 (22 - 26)	274 (208 - 365)	172 (119 - 256)
Total	84,027	0	20	30	36 (24 - 51)	20	24 (15 - 37)	40 (37 - 44)	42 (39 - 46)	356 (291 - 437)	268 (202 - 362)
**Kathmandu Medical College and Teaching Hospital**											
<2 years	7857	0	0	1	8 (0 - 71)	1	8 (0 - 71)	14 (13 - 16)	16 (15 - 18)	186 (103 - 376)	251 (120 - 613)
2 - 4 years	14,235	0	1	4	28 (8 - 72)	1	7 (0 - 39)	12 (11 - 13)	13 (12 - 14)	192 (104 - 365)	227 (102 - 522)
5 - 15 years	67,268	1	13	31	46 (31 - 65)	14	20 (11 - 35)	34 (32 - 37)	36 (33 - 40)	415 (255 - 694)	423 (228 - 826)
16-25 years	115,282	1	27	78	68 (53 - 84)	28	24 (16 - 35)	41 (38 - 45)	46 (42 - 50)	959 (523 - 1799)	653 (313 - 1440)
>25 years	243,297	0	11	27	11 (7 - 16)	11	5 (2 - 8)	8 (7 - 9)	9 (8 - 10)	149 (98 - 232)	130 (76 - 230)
Total	448,052	2	52	141	31 (26 - 37)	54	12 (9 - 16)	20 (19 - 22)	23 (21 - 25)	359 (267 - 487)	330 (230 - 480)
**Aga Khan University Hospital**											
<2 years	31,851	5	7	14	43 (24-74)	12	38 (19 - 66)	65 (60 - 71)	67 (62 - 73)	358 (244 - 555)	282 (191 - 449)
2 - 4 years	68,860	7	15	25	36 (23-54)	22	31 (20 - 48)	53 (49 - 58)	56 (52 - 61)	471 (337 - 672)	371 (265 - 534)
5 - 15 years	251,289	14	35	62	25 (19-32)	49	19 (14 - 26)	33 (30 - 36)	34 (32 - 37)	401 (290 - 561)	280 (201 - 397)
16-25 years	230,001	4	19	26	11 (7 - 17)	23	10 (6 - 15)	17 (15 - 18)	17 (16 - 19)	195 (134 - 287)	152 (103 - 230)
>25 years	593,701	4	13	20	3 (2 - 5)	17	3 (2 - 5)	5 (4 - 5)	5 (5 - 5)	29 (23 - 36)	22 (18 - 28)
Total	1,175,701	33	88	137	12 (10 -14)	122	10 (9 - 12)	18 (16 - 19)	18 (17 - 20)	134 (110 - 163)	103 (85 - 126)
**Kharadar General Hospital**											
<2 years	19,941	4	24	35	175 (122 - 244)	28	139 (93 - 203)	235 (217 - 257)	241 (222 - 263)	620 (500 - 792)	628 (470 - 889)
2 - 4 years	45,879	11	49	67	146 (113 - 185)	60	130 (100 - 168)	221 (203 - 241)	225 (208 - 246)	1008 (788 - 1312)	901 (662 - 1269)

5 - 15 years	170,998	6	44	49	29 (21 - 38)	49	29 (21 - 38)	49 (45 - 53)	50 (46 - 55)	349 (270 - 457)	344 (249 - 483)
16-25 years	139,382	1	4	5	3 (1 - 8)	5	3 (1 - 8)	6 (5 - 6)	6 (6 - 6)	37 (26 - 54)	36 (24 - 57)
>25 years	311,064	1	2	3	1 (0 - 3)	3	1 (0 - 3)	2 (2 - 2)	2 (2 - 2)	8 (6 - 10)	8 (6 - 10)
Total	6,872,65	22	123	168	24 (21 - 28)	144	21 (18 - 25)	36 (33 - 39)	36 (34 - 40)	180 (154 - 210)	176 (144 - 216)
**Paratyphi**											
**Bangladesh**											
<2 years	77,958	3	5	17	22 (13 - 35)	8	10 (4 - 20)	17 (16 - 19)	23 (22 - 25)	86 (70 - 106)	81 (66 - 101)
2 - 4 years	173,878	9	23	54	31 (23 - 41)	32	18 (13 - 26)	31 (29 - 34)	43 (39 - 47)	194 (164 - 233)	188 (157 - 228)
5 - 15 years	588,070	9	37	89	15 (12 - 19)	46	8 (6 - 10)	13 (12 - 14)	19 (17 - 20)	225 (180 - 283)	208 (165 - 262)
Total	1,019,847	22	64	161	16 (13 - 18)	86	8 (7 - 10)	14 (13 - 16)	20 (19 - 22)	135 (113 - 163)	128 (107 - 154)
**Dhulikhel Hospital, Kathmandu University Hospital**											
<2 years	1,789	0	0	0	NA	0	NA	NA	NA	NA	NA
2 - 4 years	3,392	0	0	0	10 (0 - 109)	0	NA	NA	NA	NA	NA
5 - 15 years	14,746	0	1	1	9 (0 - 38)	1	9 (0 - 38)	15 (14 - 16)	16 (14 - 17)	128 (91 - 182)	114 (62 - 224)
16-25 years	17,101	0	2	3	17 (4 - 51)	2	12 (1 - 42)	20 (18 - 21)	21 (19 - 22)	144 (98 - 221)	117 (65 - 233)
>25 years	46,967	0	0	1	3 (0 - 12)	0	1 (0 - 8)	1 (1 - 1)	1 (1 - 1)	14 (11 - 19)	9 (6 - 14)
Total	84,027	0	3	6	7 (3 - 16)	4	4 (1 - 12)	7 (7 - 8)	8 (7 - 8)	61 (50 - 75)	46 (34 - 62)
**Kathmandu Medical College and Teaching Hospital**											
<2 years	7,857	0	0	1	9 (0 - 71)	0	NA	NA	NA	NA	NA
2 - 4 years	14,235	0	1	1	9 (0 - 39)	1	7 (0 - 39)	12 (11 - 13)	13 (12 - 14)	192 (104 - 365)	227 (103 - 521)
5 - 15 years	67,268	1	1	4	7 (2 - 15)	2	3 (0 - 11)	5 (5 - 6)	6 (5 - 6)	54 (36 - 84)	66 (35 - 153)
16-25 years	115,282	0	7	14	12 (7 - 20)	7	6 (2 - 13)	11 (10 - 12)	12 (11 - 13)	255 (138 - 479)	169 (79 - 381)
>25 years	243,297	0	3	7	3 (1 - 6)	3	1 (0 - 4)	2 (2 - 2)	2 (2 - 2)	40 (26 - 63)	34 (20 - 61)
Total	448,052	1	13	28	6 (4 - 9)	13	3 (2 - 5)	5 (5 - 6)	6 (5 - 6)	87 (65 - 118)	81 (56 - 118)
**Aga Khan University Hospital**											
<2 years	31,851	0	0	0	1 (0 - 12)	0	1 (0 - 12)	2 (2 - 2)	2 (2 - 2)	13 (8 - 22)	10 (6 - 18)
2 - 4 years	68,860	0	2	2	4 (0 - 10)	2	2 (0 - 10)	4 (4 - 4)	4 (4 - 5)	49 (34 - 71)	38 (26 - 56)
5 - 15 years	251,289	1	5	9	3 (2 - 7)	6	3 (1 - 5)	4 (4 - 5)	4 (4 - 5)	60 (43 - 85)	42 (30 - 60)
16-25 years	230,001	1	7	10	4 (2 - 8)	9	4 (2 - 7)	6 (6 - 7)	7 (6 - 7)	77 (53 - 114)	60 (41 - 92)
>25 years	593,701	1	6	9	2 (1 - 3)	7	1 (0 - 2)	2 (2 - 2)	2 (2 - 2)	13 (11 - 17)	10 (8 - 13)
Total	1,175,701	3	21	31	3 (2 - 4)	24	2 (1 - 3)	4 (3 - 4)	4 (3 - 4)	30 (25 - 37)	23 (19 - 29)
**Kharadar General Hospital**											
<2 years	19,941	0	0	1	4 (0 - 28)	0	2 (0 - 18)	3 (3 - 3)	3 (3 - 3)	8 (6 - 10)	8 (6 - 12)
2 - 4 years	45,879	0	0	0	1 (0 - 8)	0	1 (0 - 8)	1 (1 - 1)	1 (1 - 1)	6 (5 - 8)	6 (4 - 8)
5 - 15 years	170,998	0	0	0	0 (0 - 2)	0	NA	NA	NA	NA	NA
Total	687,265	0	1	1	0 (0 - 1)	1	0 (0 - 1)	0 (0 - 0)	0 (0 - 0)	1 (1 - 1)	1 (1 - 1)

CI: Confidence interval. NA: Not applicable.

[1] Includes laboratory cases

[2] Inpatient and outpatient cases

Example calculation: The fully adjusted typhi incidence for Kharadar General Hospital <2 can be calculated as:
[3.7 (Inpatient cases) /.59 (culture sensitivity) / .990 (inpatient consent) / .572 (differential care-seeking)] +
[24 (Outpatient cases) /.59 (culture sensitivity) / .975 (outpatient consent) / .368 (differential care-seeking)] /
19,941 (Catchment area population) *100000

---

[i] Antillon M, Saad NJ, Baker S, Pollard AJ, Pitzer VE. The relationship between blood sample volume and diagnostic sensitivity of blood culture for typhoid and paratyphoid fever: a systematic review and meta-analysis. *The Journal of Infectious Diseases*. 2018 Nov 10;218(suppl_4):S255-67.

[ii] Hanley, James A., Abdissa Negassa, Michael D. deB Edwardes, and Janet E. Forrester. "Statistical analysis of correlated data using generalized estimating equations: an orientation." *American Journal of Epidemiology* 157, no. 4 (2003): 364-375.

[iii] Vyas: Constructing socio-economic status indices: how to use principal components analysis. https://academic.oup.com/heapol/article/21/6/459/612115
